# Controlled locomotion of a droplet propelled by an encapsulated squirmer

**DOI:** 10.1140/epje/s10189-021-00018-9

**Published:** 2021-02-18

**Authors:** R. Kree, A. Zippelius

**Affiliations:** Institut für Theoretische Physik, Georg-August-Universität Göttingen, Friedrich-Hund-Platz 1, 37077 Göttingen, Germany

## Abstract

**Abstract:**

We work out the propulsion of a viscous drop which is driven by two mechanisms: the active velocity of an encapsulated squirmer and an externally applied force acting on the squirmer. Of particular interest is the existence of a stable comoving state of drop and squirmer, allowing for controlled manipulation of the viscous drop by external forcing. The velocities of droplet and squirmer, as well as the conditions for a stable comoving state are worked out analytically for the axisymmetric configuration with a general displacement of the squirmer from the center of the droplet

**Graphic abstract:**

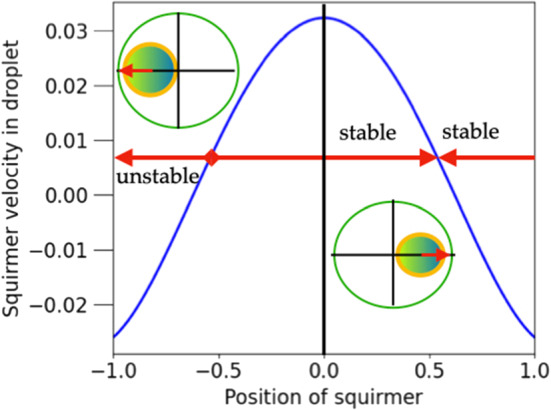

## Introduction

Self-propulsion at low Reynold’s number is a long-standing topic of research [1, 2] with a strong increase of activity in recent years [3]. It is fuelled by both, the strong desire to understand the swimming of microorganisms and cell motility as well as the need for optimal design and control of micro-robots. The latter has a wealth of important applications in the field of biomedical technology: targeted drug delivery, micro-diagnostic sensing, uptake of toxins, and many more. Most devices today are driven by magnetic fields, ultrasound and chemical reactions [4–7]; other promising candidates are biohybrid systems [8], built on sperm cells [9, 10], molecular motors or bacteria, to just mention a few. Their main advantages are biocompatibility and the supply of fuel from the surrounding fluid *in vivo*. A major challenge is their precise control by additional externally applied fields.

In the world of living microorganisms, one frequently used mechanism involves shape changes, either on the scale of the whole system such as flagella-driven motion, or confined to a thin boundary layer such as ciliary oscillations on the surface of the organism. In the simplest model of a squirmer one considers a spherical rigid body with a tangential slip velocity on its surface [2, 11]. This model is well understood and has proven to be very useful for understanding the basic features of self-propulsion. It has been generalised to Non-Newtonian fluids [12], interactions between squirmers [13, 14] and their collective behaviour [15].

Another line of approach starts from active droplets, driven by either surface tractions or force distributions in the interior of the droplet. An example for the former case is chemical reactions on the surface of the droplet, giving rise to concentration gradients and Marangoni flow [16–21]. More generally, phoretic effects have been discussed [22, 23]. The latter case of force distributions extending over the whole volume of a droplet has been realised, for example, by a collectively organised swarm of encaged magnetotactic bacteria [24], whereas encapsulated bacteria in a vesicle will cause large shape deformations and significant active surface fluctuations [25, 26]. Smooth, torque-free force distributions in the interior of a droplet [27, 28] were shown to give rise to internal rotational and mixing flow with chaotic streamlines [28], and possibly complex trajectories of the droplet [27] Collections of point forces inside the droplet [29, 30], such as stresslets, rotlets and a simple model of an autonomous biflagellate swimmer can sustain a steady comoving state of droplet and actuating device [29]. If one envisions the droplet as a drug carrier actuated by internal micro-robots, such a comoving state is desirable for robots, which would prefer to leave the droplet whenever they reach its interface.

More refined descriptions of micro-robots should take into account the finite extension and shape of the encaged devices. A simple model consists of a spherical squirmer encapsulated in a droplet. The analytic solution for a single, concentrically placed squirmer was given in [31], where also numerical solutions for eccentric configurations were provided. A steady comoving state of squirmer and droplet can be achieved if a nonuniform surface tension of the droplet is introduced [32] in addition to the active velocity on the squirmer‘s surface. The dynamics of a surfactant on the droplet‘s surface was solved perturbatively for small surface Peclet number [32]. Both papers [31, 32] consider axisymmetric configurations, resulting in translations of the droplet.

We follow up on this work and consider a simple squirmer, encapsulated in a droplet and subject to an additional external force. We focus on the propulsion velocities of the squirmer and the droplet, and we show how to calculate them analytically without having to determine the complete flow field. Our method is based on the expansion of the flow field in terms of vector solid spherical harmonics in the frame of the squirmer and a subsequent shift by the displacement $\mathbf{a}$ of the squirmer relative to the droplet‘s center. Since the computations are nontrivial for the general case, we first discuss the *axisymmetric* configurations. This allows us to explain our strategy and in itself yields interesting new results. In a subsequent publication, we will discuss configurations with a perpendicular alignment of the displacement of the squirmer and its symmetry axis. The general case is then obtained by superposition.

Our main results are simple analytical expressions for 1) the autonomous squirmer, 2) the passive particle dragged by an external force and 3) the combined system of squirmer and applied external force. For all three cases, we work out the propulsion velocity of the droplet, $v_{CM}$, and the velocity of the encapsulated particle, *U*, as a function of the system parameters, namely: the radius of the particle, its position within the droplet, the applied force,the amplitude of the slip velocity of the squirmer and the viscosity contrast between the fluids inside and outside of the droplet. We show that the droplet velocity generated by the squirmer is independent of the position of the squirmer, as long as the configuration remains axisymmetric. In contrast, the squirmer velocity shows a nontrivial dependence on $\mathbf{a}$, which is, however, not sufficient to achieve a stable comoving state of droplet and squirmer. For the passive, dragged particle, we show *i.a.* that the correction to the Stokes mobility of the encapsulated particle is regular for small particle radius, yielding further support to approaches based on point forces. Superposition of the two mechanisms, slip velocity and applied external force, can achieve a stable comoving state, where droplet and squirmer move with the same velocity in the lab frame.

## Model

We study the propulsion of a viscous droplet, which is driven by an active device in its interior. The active device is modelled as a solid squirmer with a tangential slip velocity on its surface. We first consider the force-free case and subsequently discuss a squirmer which is acted upon by an external force. In the latter case, a stationary comoving state can be achieved in which squirmer and droplet move with the same velocity.

**Fig. 1:**
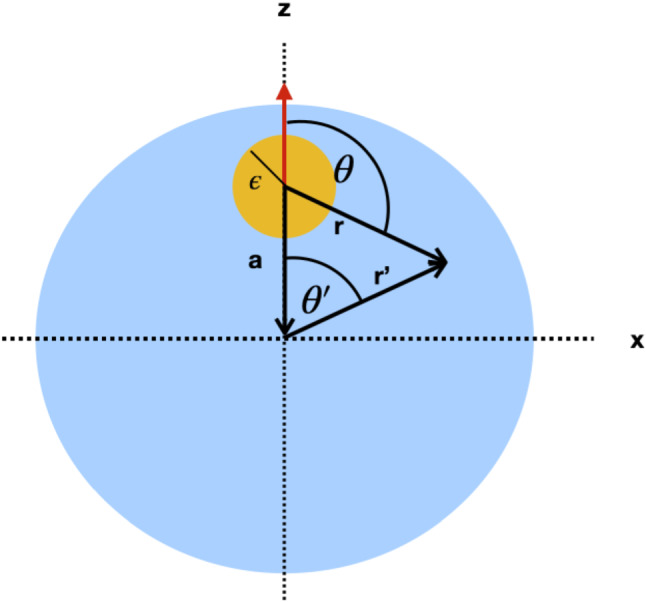
Squirmer (yellow) of radius $\epsilon$, encapsulated in a viscous drop (blue) and displaced from the centre of the drop by $-{\varvec{a}}$; the symmetry axis of the squirmer (red) is chosen to coincide with the direction of the displacement (axisymmetric configuration). In the main text, we use two different coordinate systems: ${\varvec{r}}$ denotes the position of an arbitrary point (not necessarily in the x-z plane) in a system with its origin at the centre of the squirmer; ${\varvec{r}}'$ denotes the position vector of the same point in a system with its origin at the centre of the droplet. Correspondingly, there are two systems of spherical coordinates, which represent the point in the form $(r,\theta , \phi )$ and $(r',\theta ', \phi ')$

The droplet is assumed to be spherical and consists of an incompressible Newtonian fluid with viscosity $\eta ^-$. It is immersed into an ambient Newtonian fluid of viscosity $\eta ^+$ which is at rest in the laboratory frame (LF). The two fluids are assumed to be completely immiscible. Both fluids and the squirmer are assumed to posses the same density so that the whole system is neutrally buoyant. We choose units of mass, length and time such that the density $\rho _0=1$, the droplet radius $R=1$ and the viscosity of the exterior fluid $\eta ^+=1$. We do, however, keep the notation $\eta ^+$, because some results, *e.g.* the mobility of the droplet in the exterior fluid, are more intuitive with the explicit notation.

The squirmer is modeled as a solid, rigid particle of radius $\epsilon$, shifted by $-{\varvec{a}}$ measured from the center of the droplet (see Fig.  1). The slip velocity is expanded in spherical harmonics $\chi _{lm}(\theta ,\phi )=P_{lm}(\cos {\theta })e^{im\phi }$. Here, $P_{lm}(\cos {\theta })$ denote the associated Legendre polynomials with $l=0,1,...\infty$ and $-l\le m \le l.$ In the expansion, we only keep the lowest order term, $l=1$, so that the slip velocity on the surface of the squirmer is given by

1 $$\begin{aligned} {\varvec{v}}_{slip}(\theta ,\phi )=\sum _{m=0,\pm 1}h_{m}\nabla _s\chi _{1m}(\theta ,\phi ). \end{aligned}$$

The expansion coefficients $\{h_{m}\}_{m=0,\pm 1}$ determine the strength of the 3 components of the slip velocity and $\nabla _s$ denotes the surface gradient.

The squirmer generates a flow field inside (${\varvec{v}}^-$) and outside (${\varvec{v}}^+$) of the droplet. For small Reynolds number the flow fields can be calculated from Stokes’s equation

2 $$\begin{aligned} \nabla \cdot {\varvec{\sigma }}^{\pm }=\eta ^{\pm }\nabla ^2{\varvec{v}}^{\pm }-\nabla p^{\pm }=0 , \end{aligned}$$

supplemented by the incompressibility condition $\nabla \cdot {\varvec{v}}^{\pm }=0$. The stress tensor ${\varvec{\sigma }}^{\pm }$ is given by its cartesian components $\sigma _{ij}^{\pm }=-p^{\pm }\delta _{ij}+\eta ^{\pm }(\partial _iv_j^{\pm }+\partial _jv_i^{\pm })$, with the pressure *p* determined from incompressibility.

Stokes equation has to be supplemented by boundary conditions on the surface of the squirmer, $\partial V_s$, and on the surface of the droplet, $\partial V_d$. The former determine the flow field for a point on the surface of the squirmer with position vector ${\varvec{r}}$, pointing from the center of the squirmer to the surface of the squirmer (see Fig. 1)

3 $$\begin{aligned} {\varvec{v}}^-({\varvec{r}})={\varvec{v}}_{slip}({\varvec{r}})+{\varvec{U}}. \end{aligned}$$

The velocity of the squirmer is denoted by ${\varvec{U}}$. Continuity of the flow field is assumed for points on the surface of the droplet with position vector ${\varvec{r}}^{\prime }$ pointing from the centre of the droplet to the surface of the droplet (see Fig. 1)

4 $$\begin{aligned} {\varvec{v}}^-({\varvec{r}}^{\prime })={\varvec{v}}^+({\varvec{r}}^{\prime }). \end{aligned}$$

The tangential stresses are continuous on the surface of the droplet, whereas the normal stress jumps due to a homogeneous surface tension $\gamma _0$, so that

5 $$\begin{aligned} {\varvec{e}}_{r^{\prime }}\cdot ({\varvec{\sigma }}^+-{\varvec{\sigma }}^-)=2\gamma _0{\varvec{e}}_{r^{\prime }}. \end{aligned}$$

Here, ${\varvec{e}}_{r^{\prime }}={\varvec{r}}^{\prime }/|{\varvec{r}}^{\prime }|$ is the outward normal of the droplet surface. Once the flow fields ${\varvec{v}}^{\pm }$ are known, the linear velocity of the droplet can be computed [27],

6 $$\begin{aligned} {\varvec{v}}_{CM}=\frac{3}{4\pi }\int _{\partial V_d} d^2x \;({\varvec{e}}_{r^{\prime }}\cdot {\varvec{v}}^{\pm })\,{\varvec{e}}_{r^{\prime }}, \end{aligned}$$

as an integral over the droplet‘s surface, $\partial V_d$.

## Analytical solution

We briefly outline the general strategy for the analytical solution. In a first step, the internal flow, ${\varvec{v}}^-$, is expanded in an appropriate set of functions in a coordinate system with its origin at the centre of the squirmer, and then matched to the slip velocity on the squirmer‘s surface. This solution is similar to the flow field of a squirmer in unbounded space. However, it contains not only the flow fields which are regular at infinity but also those which are regular at the squirmer‘s centre and would be forbidden in unbounded space. To fulfil the boundary condition on the droplet‘s surface, these solutions are translated by ${\varvec{a}}$ to obtain an expansion around the droplet‘s centre. Thereby an infinite series of modes is generated, even though the slip velocity of the squirmer is restricted to angular momentum components $l=1$ (see Eq. 1). However, the propulsion of the droplet is completely determined by the $l=1$ component of the internal flow and can thus be computed analytically.

The general translations for arbitrary ${\varvec{a}}$ will be presented elsewhere. In the main part of the paper, we only consider translations ${\varvec{a}}$ which are parallel to the squirmer‘s symmetry axis, taken to be the z-axis. The translated interior flow and its tractions are matched to the corresponding quantities of the external flow to fulfil the boundary conditions eqs. 4 and 5. The external flow, ${\varvec{v}}^+$, is given by those solutions of the homogeneous Stokes equation which are regular at infinity. Finally, the propulsion velocity of the droplet is computed from eq. 6. We reemphasize that in this paper we focus on the linear propulsion of the droplet and postpone a discussion of the general flow field to future work.

### Solutions of Stokes equation

The surface Laplacian acting on a vector function is diagonalised by the following set of vector spherical harmonics,

7 $$\begin{aligned} {\varvec{\Psi }}^1_{lm}= & {} \nabla _s\chi _{lm}+l\chi _{lm} {\varvec{e}}_r \end{aligned}$$

8 $$\begin{aligned} {\varvec{\Psi }}^3_{lm}= & {} \nabla _s\chi _{lm}-(l+1)\chi _{lm} {\varvec{e}}_r \end{aligned}$$

9 $$\begin{aligned} {\varvec{\Psi }}^2_{lm}= & {} {\varvec{e}}_r\times \nabla _s\chi _{lm}, \end{aligned}$$

with $\chi _{lm}=P_l^m(\cos \theta ) e^{im\phi }$ and ${\varvec{e}}_r={\varvec{r}}/r$. As far as the r-dependence is concerned, the solutions of Stokes equation can be classified according to whether they are regular at the origin or regular at infinity. We expand around the centre of the squirmer and consider a bounded region so that both types of solutions have to be taken into account. A fundamental system of solutions of Stokes’ equations which are regular at the origin is given by:

$$\begin{aligned} {\varvec{u}}_{lm}^{1<}= & {} \frac{r^{l-1}}{(l+m)!} {\varvec{\Psi }}^1_{lm}\\ {\varvec{u}}_{lm}^{2<}= & {} \frac{r^{l}}{(l+m)!(l+1)} {\varvec{\Psi }}^2_{lm}\\ {\varvec{u}}_{lm}^{3<}= & {} \frac{r^{l+1}}{(l+m)!(2l+1)} ({\varvec{\Psi }}^1_{lm}+\frac{2l}{(l+1)(2l+3)} {\varvec{\Psi }}^3_{lm}) . \end{aligned}$$

The corresponding velocities which are regular at infinity are given by:

$$\begin{aligned} \mathbf {u}_{lm}^{1>}= & {} \frac{(l-m)!}{r^{l+2}} {\varvec{\Psi }}^3_{lm}\\ {\varvec{u}}_{lm}^{2>}= & {} -\frac{(l-m)!}{lr^{l+1}} {\varvec{\Psi }}^2_{lm}\\ {\varvec{u}}_{lm}^{3>}= & {} \frac{(l-m)!}{r^l(2l+1)} (-{\varvec{\Psi }}^3_{lm} +\frac{2(l+1)}{l(2l-1)} {\varvec{\Psi }}^1_{lm}) . \end{aligned}$$

In the following, we specialise to a squirmer whose slip velocity only contains $l=1$ and $m=0$. This simplifies the discussion enormously because the generated flow only has components for $l=1$ and $m=0$. Hence, we can simplify the notation,

10 $$\begin{aligned} {\varvec{u}}^{1<}= & {} {\varvec{\Psi }}^1 \end{aligned}$$

11 $$\begin{aligned} {\varvec{u}}^{3<}= & {} \frac{r^2}{3} ({\varvec{\Psi }}^1+\frac{1}{5} {\varvec{\Psi }}^3) \end{aligned}$$

12 $$\begin{aligned} {\varvec{u}}^{1>}= & {} \frac{1}{r^3} {\varvec{\Psi }}^3 \end{aligned}$$

13 $$\begin{aligned} {\varvec{u}}^{3>}= & {} \frac{1}{3r} (-{\varvec{\Psi }}^3 +4{\varvec{\Psi }}^1), \end{aligned}$$

where it is to be understood that all ${\varvec{u}}$ and ${\varvec{\Psi }}$ refer to the $l=1$ and $m=0$ components. We have furthermore assumed that there is no chiral component in the slip velocity. The pressure follows from incompressibility and is (apart from a constant contribution) explicitly given by

14 $$\begin{aligned} p^<= 2\eta ^-r\cos {\theta } \qquad \text {and} \qquad p^>= 2\eta ^-\cos {\theta }/r^2. \end{aligned}$$

The general solution of Stokes equation ($l=1$ and $m=0$ and no chiral flow) in a bounded region is the superposition

15 $$\begin{aligned} {\varvec{v}}^-=a_1{\varvec{u}}^{1<}+a_3{\varvec{u}}^{3<}+b_1{\varvec{u}}^{1>}+b_3{\varvec{u}}^{3>}. \end{aligned}$$

The flow field outside of the droplet is given by

16 $$\begin{aligned} {\varvec{v}}^+=c_1{\varvec{u}}^{1>}+c_3{\varvec{u}}^{3>}. \end{aligned}$$

Thus the general solution involves six constants which have to be determined by the boundary conditions.

### Droplet velocity

To express the droplet velocity ${\varvec{v}}_{cm}$ by the parameters of the general solution, we insert eq. 16 into eq. 6. With ${\varvec{e}}_r\cdot {\varvec{\Psi ^1}}=\cos \theta$ and ${\varvec{e}}_r\cdot {\varvec{\Psi }}^3=-2\cos \theta$ we find

17 $$\begin{aligned} {\varvec{v}}_{CM}=v_{CM}{\varvec{e}}_z=2(c_3-c_1){\varvec{e}}_z. \end{aligned}$$

### Boundary condition on the surface of the squirmer

For the axisymmetric configuration, the squirmer velocity points in the z-direction, ${\varvec{U}}=U{\varvec{e}}_z$, and the slip velocity is given by ${\varvec{v}}_{slip}(\theta ,\phi )=-h(\cos (\theta ){\varvec{e}}_r-{\varvec{e}}_z)$. Its strength is specified by a single parameter $h=h_0$. When plugging our Ansatz into the boundary condition eq. 3 on the squirmer’s surface, we obtain two equations:

18 $$\begin{aligned} a_1+\frac{\epsilon ^2}{3}a_3+\frac{4}{3\epsilon }b_3= & {} U+\frac{2}{3} h \end{aligned}$$

19 $$\begin{aligned} \frac{\epsilon ^2}{15}a_3+\frac{1}{\epsilon ^3}b_1-\frac{1}{3\epsilon }b_3= & {} \frac{1}{3}h. \end{aligned}$$

The other four equations are provided by the boundary conditions on the droplet’s surface (eqs.4,5). However, before we can use them, we have to shift the internal flow ${\varvec{v}}^-$ by the vector ${\varvec{a}}=a{\varvec{e}}_z$.

### Translations

The boundary conditions on the surface of the droplet are conveniently expressed in terms of solid vector spherical harmonics (SVSH) which are expanded around the centre of the droplet. To obtain such an expansion, we need to know the behaviour of the SVSH under translations. The explicit calculations are a generalisation of the corresponding transformations of the solid scalar spherical harmonics, which are given in ref. [33]. For the special case under discussion, the transformation is easily worked out by hand. We, therefore, postpone the general transformation of the SVSH to a separate publication. and instead demonstrate the procedure for an example, the function ${\varvec{u}}^{3<}({\varvec{r}}^{\prime })={\varvec{u}}^{3<}({\varvec{r}}-a{\varvec{e}}_z)$. We first rewrite the function in cartesian coordinates: $5{\varvec{u}}^{3<}({\varvec{r}})=2r^2{\varvec{e}}_z-z{\varvec{r}}.$ The translation can then be done explicitly, yielding

20 $$\begin{aligned} 5 {\varvec{u}}^{3<}({\varvec{r}}-a{\varvec{e}}_z) = \begin{pmatrix} (z-a)x\\ (z-a) y\\ (z-a)^2 \end{pmatrix} -2 \begin{pmatrix} (z-a)x\\ (z-a) yx^2+y^2) \end{pmatrix}.\qquad \end{aligned}$$

We reexpress this result in terms of velocities to obtain

21 $$\begin{aligned} {\varvec{u}}_{10}^{3<}({\varvec{r}}-a{\varvec{e}}_z) ={\varvec{u}}_{10}^{3<}({\varvec{r}})+\frac{a^2}{5}{\varvec{u}}^{1<}_{10}({\varvec{r}})-\frac{2a}{5}{\varvec{u}}_{20}^{1<}({\varvec{r}}).\qquad \end{aligned}$$

We have restored the indices (*l*, *m*) to show that the translation generates higher-order *l* components. Thus, even though we started with a squirmer whose slip velocity contains just $l=1$, its off-centre position gives rise to flow with higher-order *l*-components^1^. In fact, translation of ${\varvec{u}}_{10}^{3>}$ generates all *l*-components. Here, we are interested in the propulsion of the droplet, which is solely determined by the $l=1$ component of the flow. Consequently, all $l\ge 2$ can be ignored and the translated velocity fields read:

22 $$\begin{aligned} {\varvec{u}}^{1<}({\varvec{r}}-a{\varvec{e}}_z)= & {} {\varvec{u}}^{1<}({\varvec{r}}) \end{aligned}$$

23 $$\begin{aligned} {\varvec{u}}^{3<}({\varvec{r}}-a{\varvec{e}}_z)= & {} {\varvec{u}}^{3<}({\varvec{r}})+ \frac{a^2}{5}{\varvec{u}}^{1<}({\varvec{r}})+\mathcal{O}(l\ge 2)\qquad \end{aligned}$$

24 $$\begin{aligned} {\varvec{u}}^{1>}({\varvec{r}}-a{\varvec{e}}_z)= & {} {\varvec{u}}^{1>}({\varvec{r}})+\mathcal{O}(l\ge 2) \end{aligned}$$

25 $$\begin{aligned} {\varvec{u}}^{3>}({\varvec{r}}-a{\varvec{e}}_z)= & {} {\varvec{u}}^{3>}({\varvec{r}})+ \frac{a^2}{5}{\varvec{u}}^{1>}({\varvec{r}})+\mathcal{O}(l\ge 2).\qquad \end{aligned}$$

### Boundary conditions on the surface of the droplet

Given the translated velocity fields, the application of the second boundary condition (eq.4), i.e. continuity of the velocity across the droplet‘s surface, is straightforward

26 $$\begin{aligned} a_1+\frac{a_3}{3}(1+\frac{3}{5} a^2)+\frac{4}{3}(b_3-c_3)= & {} 0 \end{aligned}$$

27 $$\begin{aligned} b_1-\frac{b_3}{3}(1-\frac{3}{5} a^2)+\frac{a_3}{15}-c_1+\frac{c_3}{3}= & {} 0. \end{aligned}$$

To fulfill balance of forces on the droplet’s surface eq. 5, we need to compute the tractions ${\varvec{t}}=-p{\varvec{e}}_r + {\varvec{t}}_{vis}$ for both, the flow inside and outside of the droplet. The viscous part is obtained using the identity

28 $$\begin{aligned} {\varvec{t}}_{vis}=2\eta \frac{\partial {{\varvec{u}}}}{\partial r}+\eta {{\varvec{e}}}_r\times (\nabla \times {{\varvec{u}}}). \end{aligned}$$

Together with the pressure contribution, we find

29 $$\begin{aligned} {\varvec{t}}^{1<}= & {} 0 \end{aligned}$$

30 $$\begin{aligned} {\varvec{t}}^{3<}= & {} \frac{3\eta ^-r}{5}{\varvec{\Psi }}^3 \end{aligned}$$

31 $$\begin{aligned} {\varvec{t}}^{1>}= & {} -\frac{6\eta ^-}{r^4}{\varvec{\Psi }}^3 \end{aligned}$$

32 $$\begin{aligned} {\varvec{t}}^{3>}= & {} \frac{2\eta ^-}{r^2}({\varvec{\Psi }}^3-{\varvec{\Psi }}^1) \end{aligned}$$

in the coordinate system of the squirmer, i.e. for the untranslated velocity fields. Since the tractions are linear functions of the velocities, the transformation to the coordinate system of the droplet is easily worked out: only ${\varvec{t}}^{3>}=\frac{2\eta ^-}{r^2}({\varvec{\Psi }}^3-{\varvec{\Psi }}^1)-\frac{6a^2}{5r^4}{\varvec{\Psi }}^3$ is affected.

The above tractions are plugged into the third boundary condition eq. 5. The pressures also contain an $l=0$ component, and the pressure difference $\Delta p$, between the interior and exterior of the droplet is determined by balancing the surface tension,

33 $$\begin{aligned} \Delta p=2\gamma _0+2\eta ^- a. \end{aligned}$$

The $l=1$-components of the third boundary condition yield the last two equations by projecting on ${\varvec{\Psi }}^1,{\varvec{\Psi }}^3$ or alternatively ${{\varvec{e}}}_r,{{\varvec{e}}}_{\theta }$:

34 $$\begin{aligned} \frac{a_3}{10}-b_1-\frac{a^2}{5} b_3+\frac{c_1}{\lambda }= & {} 0 \end{aligned}$$

35 $$\begin{aligned} c_3-\lambda b_3= & {} 0. \end{aligned}$$

Here, the viscosity contrast is denoted by $\lambda =\eta ^-/\eta ^+$.

## Results

Our approach is general enough to be applied to

- an autonomous swimmer which is solely driven by the active velocity of the squirmer,
- a passive encapsulated particle which is dragged by an external force, ${\varvec{F}}_{ext}$, and thereby propels the droplet,
- a squirmer which is driven by both, an active velocity and an external force with the possibility of a stable comoving state of the droplet and encapsulated squirmer.

Global force balance has to hold in all three cases: ${\varvec{F}}_{visc}+{\varvec{F}}_{ext}={\varvec{0}}$. We choose the external force parallel to the symmetry axis of the squirmer ${\varvec{F}}_{ext}=-f{\varvec{e}}_z$ to preserve the axisymmetric configuration. The total viscous force ${\varvec{F}}_{visc}$ can be expressed as an integral of the tractions over the surface, $\partial V(R)$, of a large sphere ($R\gg 1$)

36 $$\begin{aligned} {\varvec{F}}_{visc}=\lim _{R\rightarrow \infty } \int _{\partial V(R)} d^2 x \;{\varvec{t}}^+. \end{aligned}$$

The only flow term contributing to this expression is $c_3{\varvec{u}}^{3>}$ which is $\sim 1/r$, so that

37 $$\begin{aligned} {\varvec{F}}_{visc}= -8\pi \eta ^+ c_3{\varvec{e}}_z. \end{aligned}$$

Hence, force balance determines the coefficient $c_3$, which has to vanish for the force free, autonomous swimmer.

The propulsion velocities ${\varvec{U}}$ and ${\varvec{v}}_{CM}=2(c_3-c_1){\varvec{e}}_z$ (see eq. 17) are obtained from a linear system of 6 equations (18, 19, 26, 27, 34, 35), which contain 6 unkowns ($a_1, a_3, b_1, b_3, c_1$ and *U*). From eq. 35$c_3=\lambda b_3$, reducing the system to 5 equations for 5 unknowns. The complete solution is given in Appendix A. Here, we focus on the exact analytic expressions for the propulsion velocities ${\varvec{U}}$ of the particle and ${\varvec{v}}_{CM}$ of the droplet as functions of the system parameters $f, h, \eta ^\pm , a,$ and $\epsilon$. First, we discuss the squirmer, then we consider a passive particle dragged by an external force and finally we combine the two results to study the propulsion of a squirmer, which is steered by an applied force. Note that the possible values of $\epsilon$ and *a* are restricted by the condition $|a| + \epsilon \le 1$ to ensure that the encapsulated particle does not cross the interface.

### Squirmer without external force

If no external force is present, force balance implies $c_3=b_3=0$. From ${\varvec{v}}_{CM}=-2{\varvec{e}}_zc_1=v_{CM}{\varvec{e}}_z$ and eq. 46 we get the droplet velocity

38 $$\begin{aligned} v_{CM}= -\frac{2h}{3}\frac{5\lambda \epsilon ^3}{2\epsilon ^5(\lambda -1) +3\lambda +2}. \end{aligned}$$

Note that this velocity is independent of the squirmer position $a{\varvec{e}}_z$. For the concentric configuration, it has already been derived in ref. [31]. The propulsion velocity vanishes proportional to the squirmer volume for $\epsilon \rightarrow 0$. For $\epsilon =1$ we recover the velocity $U=U_0=-2h/3$ of a squirmer in an unbounded fluid.

**Fig. 2:**
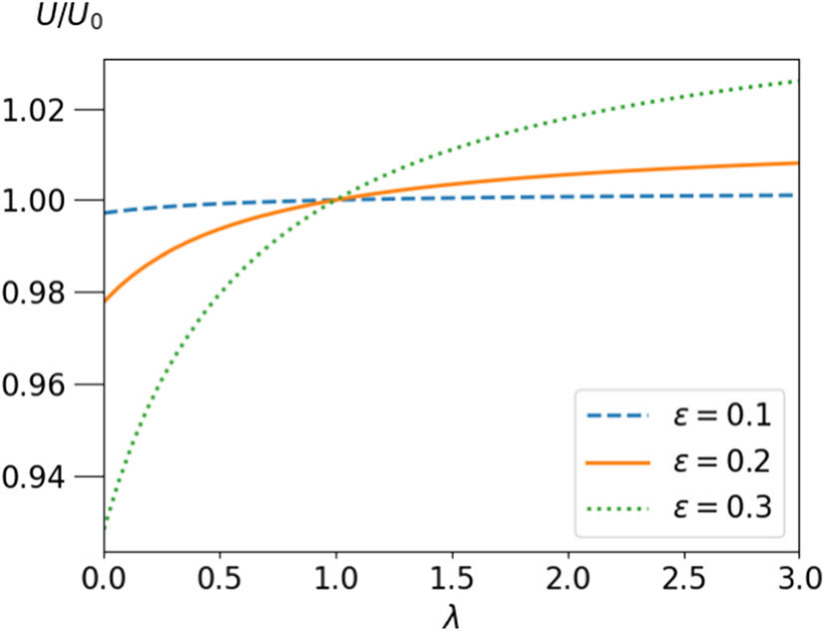
Velocity of the squirmer *U* relative to its free value as a function of the viscosity contrast $\lambda$ for 3 values of the squirmes‘ radius $\epsilon$ and $a=0.5$

Inside the droplet the squirmer velocity is given by

39 $$\begin{aligned} \frac{U}{U_0} =\frac{ 3\lambda +2 - \epsilon ^3(\lambda -1)[3\epsilon ^2 - 3 a^2 -5]}{2 \epsilon ^{5}( \lambda - 1) + 3 \lambda + 2}. \end{aligned}$$

For the special case of a concentric configuration, this expression coincides with the results of ref. [31]. For a squirmer that fills the droplet completely, i.e. for $\epsilon =1, a=0$, eq. 39 predicts $U=U_0$.

The $\lambda$-dependence of *U* is shown in Fig. 2 for $a=0.5$ and for 3 values of the squirmes‘ radius $\epsilon$. For $\lambda =1$ the velocity *U* reduces to that of the free squirmer for all values of $\epsilon$. If the fluid inside the droplet is more (less) viscous than the ambient fluid, the propulsion velocity is increased (decreased) by the encapsulation in comparison to a free squirmer.

**Fig. 3:**
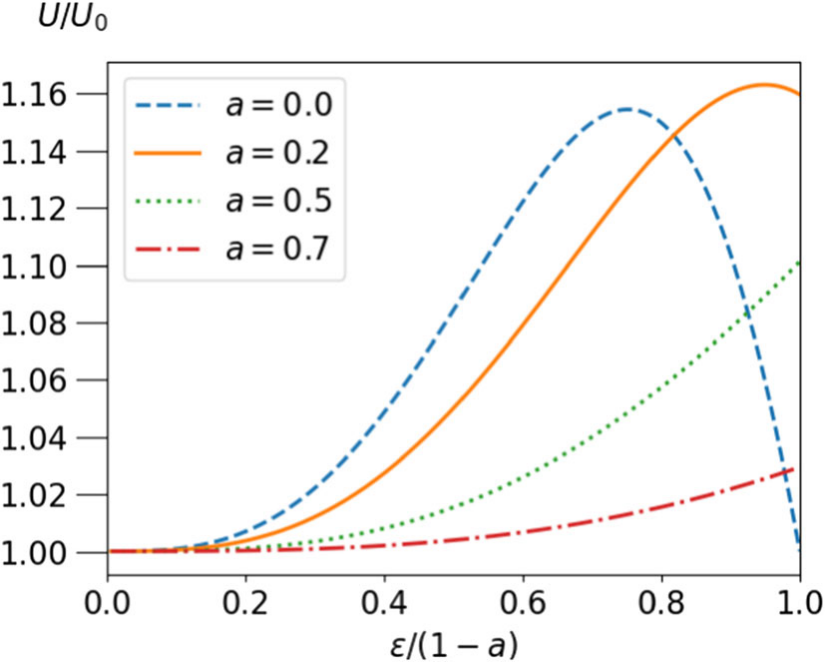
Velocity of the squirmer *U* relative to its free value as a function of the radius of the squirmer $\epsilon /(1-a)$, scaled by $1-a$, so that the abcissa varies between zero and one

The typical $\epsilon$-dependence of *U* for different positions $a{\varvec{e}}_z$ is shown in Fig. 3 versus a scaled particle size $\epsilon /(1-a)$, which ranges from 0 to 1 for all positions $a{\varvec{e}}_z$. For $a=0$ the velocity passes through a maximum in accordance with the result of ref. [31]. This maximum persists for small *a* and the maximal velocity rises beyond the value for $a=0$, until (for $\epsilon$ larger than $\approx 0.25$) the maximum reaches the boundary of possible *a*-values.

In many applications, one is interested in a stationary state in which droplet and squirmer move with the same velocity. For the autonomous swimmer, the two velocities only coincide for $\epsilon =1, a=0$. As the squirmer radius approaches 1, its velocity in the comoving frame becomes $u_{sq}=(U-v_{CM}) = 3 U_0 (1-\epsilon ) + \mathcal{{O}}(1-\epsilon )^2$. Consequently, there is no stationary position of the squirmer in a reference frame comoving with the droplet for $\epsilon <1$. Hence, an additional steering mechanism is required to achieve such a stationary state. An inhomogeneous surface tension of the droplet was suggested in ref. [32]. Here, we consider external forcing with the advantage of easy control.

### Particle dragged by an external force

To model a passive particle we choose $h=0$ and find for the droplet velocity

40 $$\begin{aligned} v_{CM}= \frac{f}{12\pi \eta ^+}\frac{3a^2- 4\epsilon ^5(\lambda -1) + 5\epsilon ^2- 3(2\lambda +3)}{2\epsilon ^5(\lambda -1)+3\lambda +2}. \end{aligned}$$

**Fig. 4:**
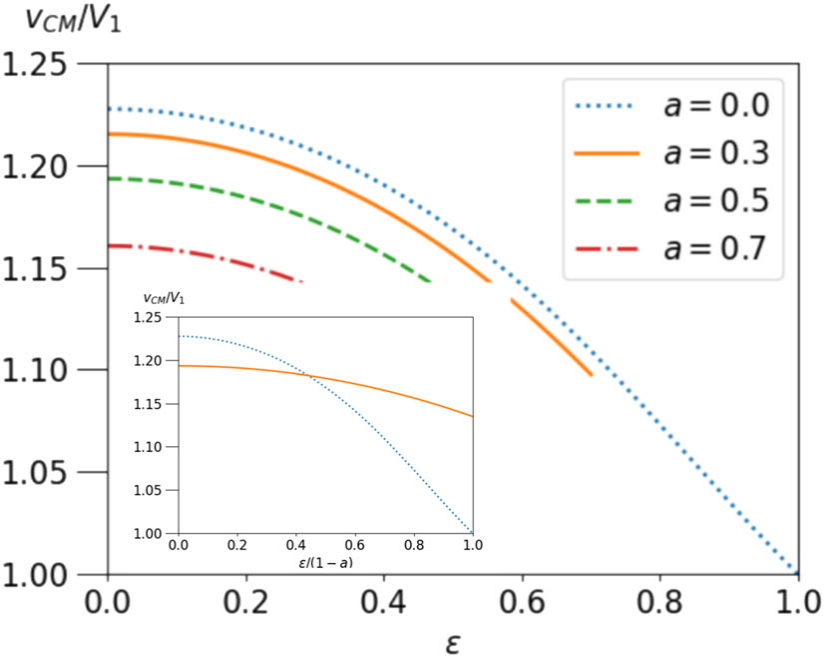
Velocity $v_{CM}$ of the droplet, relative to the Stokes value $V_1$ as a function of particle radius $\epsilon$ for $\lambda =3$ and several values of *a*. Inset: $V_{CM}/V_1$ for $a=0$ and 0.3 *vs.* scaled particle radius

For $\epsilon \rightarrow 0$ this reproduces the result for a point force in a droplet derived in our previous work [29, 34]. Corrections for small $\epsilon$ are regular in $\epsilon$ starting with terms $O(\epsilon ^2)$. For a droplet-filling particle , i.e. $\epsilon =1,a=0$ we recover the classical Stokes law $v_{CM}=V_1= -\mu _1 f$ with mobility $\mu _1=1/(6\pi \eta ^+)$ (droplet radius $R=1$ in our units). The ratio $v_{CM}/V_1$ is shown in Fig. 4 versus particle radius for $\lambda =3$ and different values of *a*. Deviations from the classical Stokes law are strongest for small $\epsilon$ and small *a*.

The particle velocity takes on the form

41 $$\begin{aligned} U&= \frac{f}{6\pi \epsilon \eta ^-}\nonumber \\&\quad \,\frac{(\lambda -1)\epsilon \Big [(9/10) a^{4} -2\epsilon ^5(\lambda -1) - (9/2) \epsilon ^4 + 5\epsilon ^2 -(3/2)(2\lambda +3)\Big ]-3\lambda -2 }{2\epsilon ^{5}(\lambda -1) + 3\lambda +2}. \end{aligned}$$

For $\lambda \rightarrow 1$ it reduces to the classical Stokes law $U=V_0=-f \mu _0$ with the free mobility $\mu _0=1/(6\pi \eta ^-\epsilon )$ corresponding to the fluid inside the droplet. The mobility ratio $U/V_0$ is regular for small epsilon and becomes $U/V_0 = 1+\mathcal{{O}}(\epsilon ).$ These findings further support approaches that model the flow field of a dragged particle by its leading multipole, i.e. the point force.

**Fig. 5:**
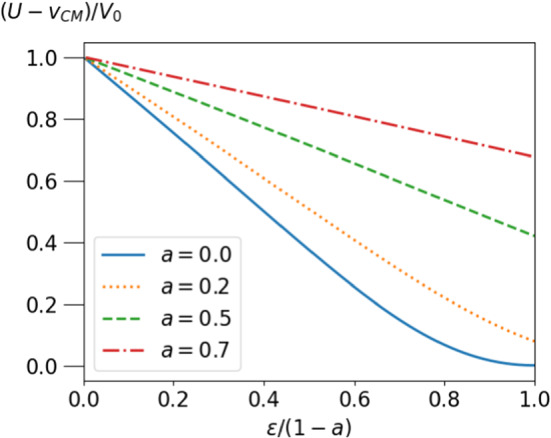
Velocity difference of dragged particle and droplet $u_p=(U-v_{CM})/V_0$ for $\lambda =3$, as a function of scaled particle radius $\epsilon (1-a).$ Only if the particle fills the droplet completely, do we observe equal velocities of droplet and particle

In Fig. 5 the relative velocity $u_p=U-v_{CM}$ is shown for $\lambda =3$ and different values of *a*. For a droplet-filling particle, i.e. $\epsilon =1, a=0$, the velocities of particle and droplet both become equal to $V_1$ for all $\lambda$. For particles with radii close to 1 the expansion of eq. 40 and eq. 41 reveals that $u_p= 3V_0(1-\epsilon )^2/2 + \mathcal{{O}}(1-\epsilon )^3$. Thus, a dragged particle cannot give rise to a stationary comoving state of droplet and particle, just like the autonomous squirmer. However, the relative velocity $u_p$ vanishes quadratically with $(1-\epsilon )$ for a dragged particle, whereas it vanishes linearly for $u_{sq}$, as shown in the previous subsection.

**Fig. 6:**
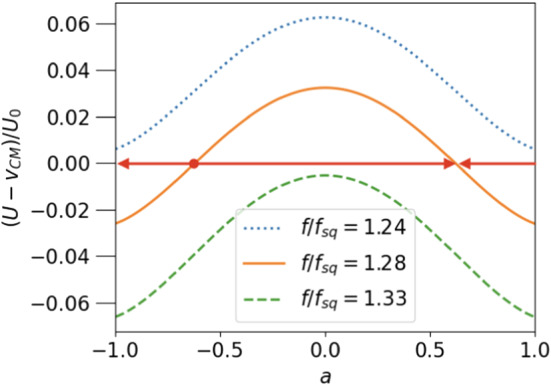
Velocity of the dragged squirmer in the frame comoving with the droplet vs. position on the z-axis. The arrows indicate the direction of squirmer motion within the droplet. The situation corresponds to viscosity contrast $\lambda =3$, other parameters are given in the text

### Steering a squirmer in a droplet by an external force

Given the propulsion properties for each of the mechanisms separately, we can now use the superposition principle to discuss a dragged squirmer. We simply add the results of eq. 38 and eq. 40 to obtain the velocity of a droplet propelled by a dragged squirmer, and eq. 39 and eq. 41 to get the velocity of this squirmer. In general, the squirmer will move in the frame comoving with the droplet and it will produce a flow, which not only propels the droplet but also changes its shape. This makes it difficult to obtain exact equations of motion for the coupled particle-droplet system. We assume that the viscous stresses on the surface of the droplet are small as compared to the surface tension. In other words, the capillary number should be small enough, so that the droplet’s shape remains nearly spherical. As an idealisation we assume a constant spherical shape and follow the calculated snapshot positions $a(t){\varvec{e}}_z$ of the particle with time. Within this approximation, we want to illustrate that it is possible to keep the squirmer stationary in the comoving frame by an appropriately balanced external force. To compare the effects of a steering force *f* with an active slip velocity $2h/3=-U_0$, we introduce the force scale $f_{sq}=U_0/\mu _0$, i.e. the external force necessary to propel a passive particle with the velocity of a squirmer in an unbounded fluid.

The appearance of stationary points of the particle within the droplet is illustrated in Fig. 6, where the relative velocity $(U-v_{cm})/U_0$ of a squirmer of radius $\epsilon =0.2$ is plotted against *a* for three positive values of $f/f_{sq}$. The undragged squirmer would move in $+z$ direction ($U_0 >0$), and the external force, $-f{\varvec{e}}_z$ pulls it in the opposite direction. The motion of the particle along the z-axis leads to two stationary points comoving with the droplet as shown in Fig. 6 . The fixpoint at positive (negative) *a* is stable (unstable) against fluctuations in the direction of propulsion, as can be read off from Fig. 6. As indicated by the arrows, the squirmer moves to the right (left) for positive (negative) values of relative velocity.

**Fig. 7:**
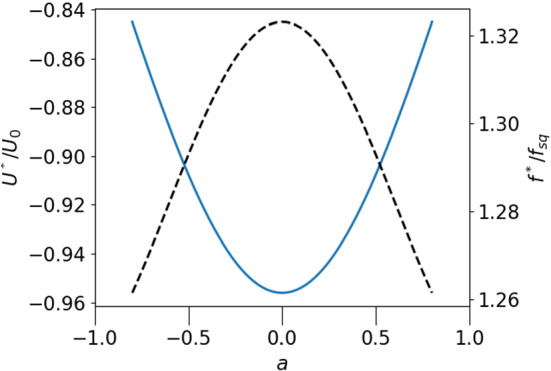
Force, $f^*/f_{sq}$, needed to achieve a stationary state of droplet and squirmer as a function of *a* (dashed line, right axis); squirmer velocity $U^*=v^*_{CM}$ in the stationary state as a function of *a* (solid line, left axis); parameters chosen: $\epsilon =0.2, \lambda =3$

**Fig. 8:**
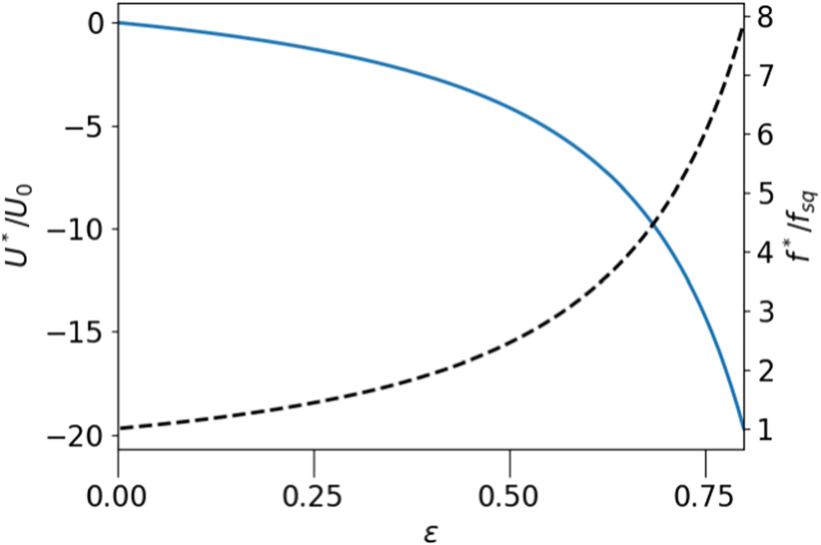
Force, $f^*/f_{sq}$ (dashed line, right axis), and squirmer velocity, $U=v_{CM}$ (solid line, left axis), as a function of $\epsilon$ (for $a=0, \lambda =3$); both functions diverge in the limit $\epsilon \rightarrow 1$;

A balancing force $f^*(\epsilon , a, \lambda , h)$, which keeps the particle at a stable fix point, can be found for *all* values of the parameters. The fix point condition reads: $U^*=v^*_{CM}$ or $u_{sq}+u_p=0.$ Since $u_{sq}\propto h$ is proportional to the active velocity and $u_{p}\propto f$ is proportional to the external force, the fix-point condition determines the ratio *f*/*h*.

To derive an explicit equation for the fix point value of the external force, $f^*$, given the active velocity *h*, we rewrite the relative velocities as $u_{sq}=U_0\hat{u}_{sq}$, $u_p=V_0\hat{u}_p$. The fix point condition then takes on the form

42 $$\begin{aligned} \frac{f^*}{f_{sq}}= \frac{\hat{u}_{sq}(\epsilon , a, \lambda )}{\hat{u}_p(\epsilon ,a,\lambda )}, \end{aligned}$$

which immediately gives $f^*(\epsilon , a,\lambda , h)$. The a-dependence of the balancing force $f^*/f_{sq}$ is shown in Fig. 7 (for $\epsilon =0.2$ and $\lambda =3$), together with the corresponding velocity of the drop, $U^*$, at the stationary point. Fixing the particle at the center of the drop requires the largest force and also produces the largest propulsion velocity. If the radius of the particle is increased, $f^*$ and $U^*$ grow significantly. This is shown in Fig. 8 for $a=0$. Both quantities even diverge for $\epsilon \rightarrow 1$, which can be seen by inserting the limiting behaviour of $u_{sq}$ and $u_p$, derived in subsections 4.1 and 4.2 into eq. 42. This yields $1/f^*=(1-\epsilon )/2 +\mathcal{{O}}((1-\epsilon )^2)$. From Fig. 8 one can read off, for example, that a droplet encapsulating an active spherical particle of half of the droplet’s radius can be used to propel it via an external force with velocities, which exceed the active squirmer’s velocity by a factor of 10 and more, while the steered squirmer stays stationary inside the droplet.

## Conclusions and outlook

As discussed in the introduction, many biomedical applications make use of tiny, micrometre-sized compartments which can be loaded and moved in a controlled way. We have shown here that a viscous drop with an encapsulated squirmer provides one possible realisation of such a device. The active velocity of the squirmer can propel the droplet with, however, different velocities of droplet and squirmer. An additionally applied external force can then be adjusted to produce a stationary comoving state in which both, the droplet and the squirmer, move with the same velocity. Our analysis is fully analytic, so that the dependence on squirmer position, radius, viscosity contrast, *etc*. is explicit.

Here, we have presented results only for the axisymmetric configuration. A second paper deals with displacements of the squirmer perpendicular to its symmetry axis, i.e. the transverse configurations. Several other extensions can be considered within the general framework. Examples are a chiral component in the active velocity and higher-order $l-$components. Of particular interest is $l=2$, allowing to distinguish between pullers and pushers. A squirmer with exclusively $l=2$ components does not move in unbounded space but is expected to be suitable for self-propulsion if encapsulated in a droplet.

## Coefficients of the full $l=1$ flow

For the squirmer (i.e. for ${\varvec{F}}={\varvec{0}}$) the solutions of the system equations 18, 19, 26, 27, 34 with $c_3=b_3= 0$ read

43 $$\begin{aligned} a_{1}= & {} -\frac{2}{3}h(\lambda -1)\epsilon ^3\frac{3a^2+5}{2\epsilon ^{5}(\lambda -1) + 3\lambda +2} \end{aligned}$$

44 $$\begin{aligned} a_{3}= & {} \frac{2}{3}h(\lambda -1)\epsilon ^3\frac{15}{2\epsilon ^{5}(\lambda -1) + 3\lambda +2} \end{aligned}$$

45 $$\begin{aligned} b_{1}= & {} \frac{h\epsilon ^3}{3}\frac{3\lambda +2}{2\epsilon ^{5}(\lambda -1) + 3\lambda +2} \end{aligned}$$

46 $$\begin{aligned} c_1= & {} \frac{h\epsilon ^3}{3}\frac{5\lambda }{2\epsilon ^{5}(\lambda -1) + 3\lambda +2} \end{aligned}$$

Note that the only a-dependent coefficient is $a_{1}$

For the dragged particle $c_3= -f/8\pi \eta ^+ = \lambda b_3$ and we get

47 $$\begin{aligned} a_{1}= & {} -\frac{F(\lambda -1)}{60\pi \eta ^-}\nonumber \\&\frac{ (3a^2-5\!+\!5\epsilon ^2)(3a^2+5) \!-\!20\epsilon ^5(\lambda -1) \!-\! 10(3\lambda \!+\!2) }{2\epsilon ^{5}(\lambda -1) \!+\! 3\lambda +2}\nonumber \\ \end{aligned}$$

48 $$\begin{aligned} a_{3}= & {} -\frac{F(\lambda -1)}{4\pi \eta ^-}\frac{5(1-\epsilon ^2) - 3 a^2}{2\epsilon ^{5}(\lambda -1) + 3\lambda +2} \end{aligned}$$

49 $$\begin{aligned} b_{1}= & {} -\frac{F\epsilon ^2}{120\pi \eta ^-}\frac{6a^2\epsilon ^3(\lambda -1) -10\epsilon ^3(\lambda -1) - 15\lambda -10}{2\epsilon ^{5}(\lambda -1) + 3\lambda +2}\qquad \end{aligned}$$

50 $$\begin{aligned} c_{1}= & {} \frac{F}{24\pi \eta ^+}\frac{2\epsilon ^5(\lambda -1) + 3 (\lambda -1) + 5\epsilon ^2 +3a^2}{2\epsilon ^{5}(\lambda -1) + 3\lambda +2} \end{aligned}$$

## Footnotes

1 For the axisymmetric configuration, no $m=\pm 1$ are generated.
